# A robust permutation test for the concordance correlation coefficient

**DOI:** 10.1002/pst.2101

**Published:** 2021-02-17

**Authors:** Alan D. Hutson, Han Yu

**Affiliations:** ^1^ Department of Biostatistics and Bioinformatics Roswell Park Comprehensive Cancer Center Buffalo New York USA

**Keywords:** measures of agreement, non‐normal, small sample, studentization

## Abstract

In this work, we developed a robust permutation test for the concordance correlation coefficient (*ρ*_*c*_) for testing the general hypothesis *H*_0_ : *ρ*_*c*_ = *ρ*_*c*(0)_. The proposed test is based on an appropriately studentized statistic. Theoretically, the test is proven to be asymptotically valid in the general setting when two paired variables are uncorrelated but dependent. This desired property was demonstrated across a range of distributional assumptions and sample sizes in simulation studies, where the test exhibits robust type I error control in all settings tested, even when the sample size is small. We demonstrated the application of this test in two real world examples across cardiac output measurements and endocardiographic imaging.

## 1 INTRODUCTION

Measurement of agreement is an essential task in biology and medicine. The often encountered question is whether measurements by two different methods on the same samples produce essentially the same results. For example, it may be of interest to evaluate whether a new assay can reproduce the results of a traditional gold‐standard assay for measuring tumor biomarkers in serum, or whether two pathologists have the same ratings on a set of samples for a cancer diagnosis. The measurement of agreement consists of two aspects: accuracy and precision. Accuracy pertains to whether the observed value agrees with the true value systematically, while precision measures the extent to which the observed values conform.^1^

Specific agreement measurements have been designed for different types of data. For categorical data with two levels, McNemar's test is typically used to assess the systematic difference between two measurements. A significant test result would suggest two measurements deviate in a systematic manner. Cohen's *κ* is a single value measurement for agreement between categorical variables, which is defined as the difference between observed and expected agreement by chance.^2^ The approach can be extended to ordinal data with more than two categories by using appropriate weighting schemes.^3^ For continuous data, the paired‐sample *t*‐test can be used to measure the systematic differences between paired observations. The Bland and Altman diagram plots the difference between two measurements against their means, so as to visualize the pattern and extent of agreement relative to the overall variation.^4^ The intraclass correlation coefficient (ICC) can be used as single value measurement of agreement, which represents the between‐pair variance as a proportion of the total variance of the observations.^5^

Lin (1989)^6^ proposed the concordance correlation coefficient (CCC), which is a widely used and highly cited agreement index between pairs of continuous measurements. Several R packages are available for its calculation, such as DescTools, agRee and cccrm.^7^, ^8^, ^9^ The CCC evaluates the agreement between two readings by measuring the variation of their linear relationship from the 45° line through the origin (the concordance line). It can be expressed as the product of the Pearson correlation coefficient *ρ*, which measures precision, and a measurement of accuracy, which is a function of the means and standard deviations. This is an advantage of the CCC over other measurements since it evaluates precision and accuracy simultaneously in a single measure. The CCC has also been extended to be modeled as a function of covariates^10^, ^11^ and a measure of overall agreement among multiple raters.^12^ Nonparametric tests have also been developed to assess the multi‐rater agreements based on the CCC.^13^

The CCC has become a popular tool for measuring agreement. Hypothesis testing on the CCC (*H*_0_ : CCC = CCC_0_) is important in assessing whether there is sufficient agreement between two measurements. The test is typically based on the asymptotic distribution of either ${\hat{\rho }}_{c}$ or the *Z*‐transformed statistic.^6^ It has been widely used in real world applications,^14^, ^15^, ^16^, ^17^ and has been implemented in the Stata CONCORD module.^18^, ^19^ Both asymptotic tests rely on large sample sizes and typically fail to control the Type I error at the desired level when *n* is small. Under such scenarios, permutation tests provide a strong alternative testing approach. To our knowledge, only limited work has been reported on permutation test about the CCC. Williamson et al.^20^ proposed a permutation test for the CCC for comparing whether two methods have equal agreement with the third, for example a gold standard. However, permutation tests for a point null were not a part of their work.

A common and naive mistake in terms of permutation testing about the correlation coefficient or the CCC is to perform a simple permutation test ignoring possible dependency structures, which leads to invalid inference. Since the CCC can be decomposed into the product of Pearson's correlation with a quantity measuring bias, inference about these two measurements are closely related. DiCiccio and Romano have shown that the permutation distribution of Pearson's correlation coefficient does not converge to the sampling distribution when two random variables are dependent but uncorrelated.^21^ Therefore, the type I error rate will not be controlled at the desired level. They showed that this issue can be solved by using a permutation test based on an appropriately studentized statistic.

In Section 2 we show that a naive permutation test about the CCC behaves similarly to the non‐studentized permutation test about Pearson's correlation coefficient in terms of inflated Type I error rates. To address this issue we propose a studentized statistic for the CCC following the approach of DiCiccio and Romano.^21^ More importantly, we extended the studentized permutation test to more general null hypotheses: *H*_0_ : CCC = CCC_0_. Studentized statistics have been widely used in permutation tests.^22^, ^23^ However, to our knowledge, this is the first work using studentized permutation test for the CCC. We prove theoretically that the permutation test for the CCC based on studentized statistic is asymptotically valid. In Section 3 we carry out an extensive simulation study which illustrated that studentized permutation test controls the Type I error at its nominal level even in the small sample size settings. Finally, in Section 4 we demonstrate our methodology using real world data from studies on cardiac output measurements and endocardiographic imaging.

## 2 METHODS

### 2.1 Concordance correlation coefficient

Let (*X*_1_, *Y*_1_), …, (*X*_*n*_, *Y*_*n*_) be *n* pairs of samples independently selected from a bivariate population with means *μ*_1_ and *μ*_2_, and covariance matrix

$$\Sigma =\left(\begin{array}{cc} \sigma_{1}^{2} & \sigma_{12}\\ \sigma_{21} & \sigma_{2}^{2} \end{array}\right).$$

The CCC is based on the expected value of the squared difference of two variables, *X* and *Y*, and defined as,

$$\rho_{c}=1-\frac{E\left[{\left(X-Y\right)}^{2}\right]}{\sigma_{1}^{2}+\sigma_{2}^{2}+{\left(\mu_{1}-\mu_{2}\right)}^{2}}=\frac{2\sigma_{12}}{\sigma_{1}^{2}+\sigma_{2}^{2}+{\left(\mu_{1}-\mu_{2}\right)}^{2}},$$

where −1 ≤ *ρ*_*c*_ ≤ 1. Note that the CCC can be decomposed into two parts as follows:

$$\rho_{c}=\frac{2\sigma_{12}}{\sigma_{1}^{2}+\sigma_{2}^{2}+{\left(\mu_{1}-\mu_{2}\right)}^{2}}=\mathrm{\rho C},$$

where *ρ* is the Pearson correlation coefficient, which measures linear association between *X* and *Y*, and

$$C=\frac{2\sigma_{1}\sigma_{2}}{\sigma_{1}^{2}+\sigma_{2}^{2}+{\left(\mu_{1}-\mu_{2}\right)}^{2}},$$

which is a measure of accuracy, and represents how far the best‐fit line deviates from the 45° line through origin (concordance line). The value of *ρ*_*c*_ = 1 indicates perfect agreement while the value of *ρ*_*c*_ = 0 indicates lack of agreement. It is important to note that *ρ*_*c*_ = 0 if and only if *ρ* = 0.

The null hypothesis that is of interest to most researchers in the context of testing agreement is *H*_0_ : *ρ*_*c*_ = *ρ*_*c*(0)_ and we will focus on one‐sided alternative hypothesis *H*_1_ : *ρ*_*c*_ > *ρ*_*c*(0)_. In agreement testing the test *H*_0_ : *ρ*_*c*_ ≥ *ρ*_*c*(0)_ versus *H*_1_ : *ρ*_*c*_ < *ρ*_*c*(0)_ is generally not of interest in practice. For *n* independent sample pairs (*X*_1_, *Y*_1_), …, (*X*_*n*_, *Y*_*n*_), *ρ*_*c*_ can be estimated by replacing the population quantities with the respective moment estimators such that

$$\begin{array}{c} {\hat{\rho }}_{c}=\frac{2{\hat{\sigma }}_{12}}{{\hat{\sigma }}_{1}^{2}+{\hat{\sigma }}_{2}^{2}+\left({\hat{\mu }}_{1}-{\hat{\mu }}_{2}\right)}=\hat{\rho }\hat{C}\\ \hat{C}=\frac{2{\hat{\sigma }}_{1}{\hat{\sigma }}_{2}}{{\hat{\sigma }}_{1}^{2}+{\hat{\sigma }}_{2}^{2}+{\left({\hat{\mu }}_{1}-{\hat{\mu }}_{2}\right)}^{2}}, \end{array}$$

where ${\hat{\mu }}_{1}$ and ${\hat{\mu }}_{2}$ are the sample means, ${\hat{\sigma }}_{1}^{2}$ and ${\hat{\sigma }}_{2}^{2}$ are the sample variances, for *X* and *Y*, respectively and ${\hat{\sigma }}_{12}$ is the sample covariance. Note that *ρ*_*c*_ = 0 if and only if *ρ* = 0. However, testing *H*_0_ : *ρ*_*c*_ = 0 and *H*_0_ : *ρ* = 0 are different tests in that the estimator $\hat{\rho }$ is scaled by a random variable $\hat{C}$ in ${\hat{\rho }}_{c}$ compared with $\hat{\rho }$, for the respective tests.

The test about ${\hat{\rho }}_{c}$ can be performed based on the asymptotic normal distribution of either ${\hat{\rho }}_{c}$ or using the Fisher's *Z* transformation given as,

$$\hat{Z}=\tanh^{-1}\left({\hat{\rho }}_{c}\right)=\frac{1}{2}\ln\frac{1+{\hat{\rho }}_{c}}{1-{\hat{\rho }}_{c}}.$$

The variances for each test are obtained utilizing the delta method, for example, see Lin (1989)^6^ for details of deriving the asymptotic distributions for both statistics, respectively. When the sample size is small, the Type I error is usually not well controlled, even though the *Z*‐transformed statistic converges at a faster rate to normality.^6^ We will illustrate this property in our simulation study in Section 3.

### 2.2 Background information

As noted earlier the CCC, *ρ*_*c*_, may be decomposed into two components, namely *ρ* rescaled by a non‐zero constant *C*. Therefore, the tests of *ρ*_*c*_ and *ρ* are closely related. In this section, we start by reviewing the permutation test for Pearson's correlation coefficient as developed by Diciccio and Romano.^21^ Towards this end define **G**_*n*_ to be the set of all permutations *π* of {1, …, *n*}. For testing independence between two random variables *X* and *Y*, the permutation distribution of any given test statistic *T*_*n*_(*X* ^*n*^, *Y* ^*n*^) is defined as

(1) $${\hat{R}}_{n}^{T_{n}}\left(t\right)=\frac{1}{n!}\sum_{\pi \in G_{n}}I\left\{T_{n}\left(X^{n},Y_{\pi }^{n}\right)\leq t\right\},$$

where $Y_{\pi }^{n}$ represents {*Y*_*π*(1)_, …, *Y*_*π*(*n*)_}. In this setting, the permutation **G**_*n*_ is all possible pairwise combinations between *X* ^*n*^ and *Y* ^*n*^. A level *α* one‐sided permutation test rejects if $T_{n}\left(X^{n},Y_{\pi }^{n}\right)$ is larger than the 1 − *α* quantile of the permutation distribution. The permutation test is exact when exchangeability assumptions hold, that is, the distribution of (*X* ^*n*^, *Y* ^*n*^) is invariant under the group of transformations **G**_*n*_. The test using the Pearson correlation coefficient $\hat{\rho }$ is exact when using a metric of dependence for testing the null hypothesis of independence given as

$$H_{0}:P=P_{X}\times P_{Y},$$

where *P*_*X*_ and *P*_*Y*_ are marginal distributions. The null hypothesis of independence is not equivalent to the test about zero correlation given as *H*_0_ : *ρ* = 0 with the exception of limiting assumptions such as the data are distributed as bivariate normal random variables. In other words, in the general setting two random variables can be dependent but uncorrelated. In such cases, DiCiccio and Romano^21^ have shown that, with finite fourth moments, the permutation distribution of $\hat{\rho }$ converges to *N*(0, 1), but its sampling distribution converges to *N*(0, *τ* ^2^), where

$$\tau^{2}=\frac{\mu_{22}}{\mu_{20}\mu_{02}},$$

and

$$\mu_{\mathrm{rs}}=E\left[{\left(X_{1}-\mu_{1}\right)}^{r}{\left(Y_{1}-\mu_{2}\right)}^{s}\right].$$

Thus the test will not be level *α* unless *τ* = 1. In light of this result, DiCiccio and Romano proposed a studentized correlation test statistic, which has been shown to control Type I error asymptotically at *α* when two random variables are dependent but uncorrelated.^21^ Specifically, the studentized statistic is defined as $S_{n}=\sqrt{n}{\hat{\rho }}_{n}/{\hat{\tau }}_{n}$, where

$$\begin{array}{c} {\hat{\tau }}_{n}^{2}=\frac{{\hat{\mu }}_{22}}{{\hat{\mu }}_{20}{\hat{\mu }}_{02}},\\ {\hat{\mu }}_{\mathrm{rs}}=\frac{1}{n}\sum_{i=1}^{n}{\left(X_{i}-\bar{X}\right)}^{r}{\left(Y_{i}-\bar{Y}\right)}^{s}. \end{array}$$

The permutation distribution and sampling distribution of *S*_*n*_ both converge to the standard normal distribution asymptotically. It should be noted that even though the results presented in DiCiccio and Romano^21^ are based on large sample approximations the behavior of this test for small to moderate sample sizes is quite good as born out in their simulation results.

### 2.3 Permutation concordance correlation test for *H*_0_ : *ρ*_*c*_ = 0

For the permutation test of *ρ*_*c*_ = 0, we use the same permutation scheme used for the Pearson correlation coefficient^21^ as described in Equation (1). That is, for each permutation we will randomly shuffle *Y* while keeping *X* fixed. Recall that *ρ*_*c*_ = 0 if and only if *ρ* = 0, but we also have *ρ*_*c*_ → 0 when *σ*_1_/*σ*_2_ →  + ∞ or 0, or when ∣*μ*_1_ − *μ*_2_ ∣  →  + ∞. The latter condition implies *F*_*x*_ and *F*_*y*_ have either location or scale differences or both. Therefore, for any permutation scheme, the exchangeability assumption does not necessarily hold under *H*_0_. In this section we show that the permutation test using the permutation scheme by randomly shuffling *Y*, although not exact, will be asymptotically valid if the statistic is properly studentized. On the other hand, the naive permutation test based on the statistic ${\hat{\rho }}_{c}$ defined at Section 2.1 suffers a similar deficiency to that of $\hat{\rho }$ for Pearson's correlation coefficient, and it does not generally control the Type I error at the desired level.

From the result of DiCiccio and Romano,^21^ if $E\left(X_{1}^{2}\right)<\infty ,E\left(Y_{1}^{2}\right)<\infty$ and $E\left(X_{1}^{2}Y_{1}^{2}\right)<\infty$, then under *H*_0_, $\sqrt{n}{\hat{\rho }}_{n}\to \mathrm{\tau Z}$, where *Z* ∼ *N*(0, 1). By the strong law of large numbers, we have ${\hat{C}}_{n}\to C$ almost surely. Therefore, by Slutsky's theorem, we have $T_{n}^{c}=\sqrt{n}{\hat{\rho }}_{n}{\hat{C}}_{n}\to \mathrm{\tau CZ}$ in distribution under *H*_0_.

Proposition 1 *Let C*_*n*_*and T*_*n*_*be functions of a sequence of i.i.d. random variables X*_*n*_*. If C*_*n*_ → *C almost surely, and*

$$\lim_{n\to \infty }\underset{t\in R}{\sup}|{\hat{R}}_{n}^{T_{n}}\left(t\right)-F\left(t\right)|=0$$

*for almost every sequence of X*_*n*_ *, where F(t) is the CDF of Z, then for statistic* $T_{n}^{\prime }=C_{n}T_{n}$ *, we have*

$$\lim_{n\to \infty }\underset{t\in R}{\sup}|{\hat{R}}_{n}^{T_{n}^{\prime }}\left(t\right)-F^{\prime }\left(t\right)|=0,$$

*under H*_0_ : *ρ*_*c*_ = 0*, where F* ^′^(*t*) *is the CDF of Z* ^′^ = *CZ*.

Note that for a given sample, $\hat{C}\left(X^{n},Y_{\pi }^{n}\right)$ remains constant for any *π*, and ${\hat{C}}_{n}\to C$ almost surely. From Theorem 2.1 of DiCiccio and Romano^21^ it follows that

$$\lim_{n\to \infty }\underset{t\in R}{\sup}|{\hat{R}}_{n}^{T_{n}}\left(t\right)-F\left(t\right)|=0.$$

Therefore, by Proposition 1, we have,

$$\lim_{n\to \infty }\underset{t\in R}{\sup}|{\hat{R}}_{n}^{T_{n}^{c}}\left(t\right)-\Phi_{C}\left(t\right)|=0$$

almost surely, where Φ_*C*_ is the CDF of *N*(0, *C* ^2^). We can see that the sampling and permutation distributions of *T*_*n*_ converge to the same distribution only when *τ* = 1, thus the permutation test will not guarantee Type I error control at level *α* under general scenarios. On the other hand, the permutation test will be asymptotically valid when it is based on a studentized statistic. Towards this end we have the following:

Theorem 1 *Let* (*X*_*n*_, *Y*_*n*_) *be a sequence of i.i.d. random variables. Suppose* $E\left(X_{1}^{4}\right)<\infty$ *and* $E\left(Y_{1}^{4}\right)<\infty$ *, and define the studentized statistic*

$$S_{n}^{c}=\sqrt{n}{\hat{\rho }}_{n}{\hat{C}}_{n}/{\hat{\tau }}_{n},$$

*then we have*

$$\lim_{n\to \infty }\underset{t\in R}{\sup}|{\hat{R}}_{n}^{S_{n}^{c}}\left(t\right)-\Phi_{C}\left(t\right)|=0,$$

*almost surely under H*_0_ : *ρ*_*c*_ = 0*, where* Φ_*C*_ *is the CDF of N*(0, *C* ^2^).

The proof of Theorem 1 follows directly from the fact that *C*_*n*_ is constant under permutations, Theorem 2.2^21^ and Proposition 1. Therefore, both the sampling distribution and permutation distribution of $\sqrt{n}S_{n}^{c}\left(X^{n},Y^{n}\right)$ converge to the corresponding quantiles of a *N*(0, *C* ^2^) distribution, which in turn proves the test has asymptotic Type I error control at level *α*. Note that although $\hat{C}$ remains constant under the proposed permutation scheme, the tests on the Pearson's correlation coefficient and the CCC are distinctly different tests.

### 2.4 Permutation concordance correlation test for *H*_0_ : *ρ*_*c*_ = *ρ*_*c*(0)_

In a more general scenario, we may be interested in testing *H*_0_ : *ρ*_*c*_ = *ρ*_*c*(0)_ versus *H*_0_ : *ρ*_*c*_ > *ρ*_*c*(0)_. This corresponds to a non‐zero correlation under *H*_0_, which cannot be tested by a conventional permutation test. In order to bring this test into the above framework, we rely on a statistic based on a de‐correlated sample.

First, we obtain an estimated correlation under the *H*_0_:

$${\hat{\rho }}_{0}=\rho_{c\left(0\right)}/\hat{C},$$

which converges almost surely to *ρ*_0_ when *H*_0_ is true. We can standardize the original observations by

$$U_{i}^{\prime }=\frac{X_{i}-\bar{X}}{S_{X}},V_{i}^{\prime }=\frac{Y_{i}-\bar{Y}}{S_{Y}},$$

such that the new variables will have zero means and unit standard deviations, which will then be de‐correlated by

$${\left(U,V\right)}^{T}=A\left({\hat{\rho }}_{0}\right){\left(U^{\prime },V^{\prime }\right)}^{T}.$$

In this equation, the matrix **A**(⋅) is defined as

$$A\left(x\right)=\left(\begin{array}{cc} 1 & 0\\ \frac{-x}{\sqrt{1-x^{2}}} & \frac{1}{\sqrt{1-x^{2}}} \end{array}\right),$$

which satisfies

$$A\left(\rho \right)\left(\begin{array}{cc} 1 & \rho \\ \rho & 1 \end{array}\right)A{\left(\rho \right)}^{T}=I_{2}.$$

It is straightforward to show that $\hat{\rho }\left(U,V\right)\to 0$ under *H*_0_. The test statistic can then be defined based on *U* and *V*,

$$S_{n}^{c}=\sqrt{n}{\hat{\rho }}_{n}\left(U,V\right){\hat{C}}_{n}/{\hat{\nu }}_{n},$$

where ${\hat{\rho }}_{n}\left(U,V\right)$ is the sample Pearson correlation of *U* and *V*, and $\nu_{n}^{2}$ is the large sample variance of ${\hat{\rho }}_{n}\left(U,V\right)$. By large sample theory, it can be readily shown that $\sqrt{n}{\hat{\rho }}_{n}\left(U,V\right)\to N\left(0,\nu_{n}^{2}\right)$. Therefore, we have

$$S_{n}^{c}\to N\left(0,C^{2}\right).$$

Obviously, the pair (*U*, *V*) is asymptotically uncorrelated under *H*_0_, which means they are also asymptotically exchangeable under normality. Therefore, under the framework by DiCiccio and Romano,^21^ it is legitimate to obtain the permutation distribution of $S_{n}^{c}$ by randomly shuffling *V*. Specifically, for each permutation we will calculate the statistic as

$$S_{n}^{c}\left(U,V_{\pi }\right)=\sqrt{n}{\hat{\rho }}_{n}\left(U,V_{\pi }\right){\hat{C}}_{n}.$$

By Theorem 1, we can see that the permutation distribution of $S_{n}^{c}$ will converge to *N*(0, *C* ^2^) when *ρ*(*U*, *V*) = 0, a condition will be satisfied asymptotically.

The computation of $S_{n}^{c}$ relies on the estimation of $\nu_{n}^{2}$, of which the analytical form is very difficult to derive. The commonly used bootstrap method yields poor variance estimation in our case (data not shown). This is likely due to the standardization step which tends to be unstable when there are many duplicates during the resampling, which can be more severe when the sample size is small. On the other hand, the jackknife method provides a robust estimation. Conventionally, the jackknife procedure calculates $S_{n\left(i\right)}^{c}$ for *i* = 1, …, *n*, where $S_{n\left(i\right)}^{c}$ is the statistic with (*X*_*i*_, *Y*_*i*_) left out, and the variance can be estimated by ${\hat{\nu }}_{n}^{2}=\left(n-1\right)\hat{V}\mathrm{ar}\left(S_{n\left(i\right)}^{c}\right)$. It should be noted that this variance is estimated under $\rho_{c}={\hat{\rho }}_{c}$. However, for hypothesis testing the variance needs to be estimated under *ρ*_*c*_ = *ρ*_*c*(0)_. It is obvious that $\nu_{n}^{2}$ depends on *ρ*_*c*_ and such discrepancy may lead to a reduced power. To solve this issue, we used a surrogate distribution approach used in the work by Hutson.^24^

The surrogate data is obtained by a de‐correlation operation followed by a re‐correlation based on the correlation expected under *H*_0_ which is ${\hat{\rho }}_{0}$. Specifically, let $U_{i}^{\prime }$ and $V_{i}^{\prime }$ be the standardized observations. They will be transformed as below

$${\left(X_{i}^{\prime },Y_{i}^{\prime }\right)}^{T}=\left(\begin{array}{cc} S_{X} & 0\\ 0 & S_{Y} \end{array}\right)B\left({\hat{\rho }}_{0}\right)A\left(\hat{\rho }\right){\left(U_{i}^{\prime },V_{i}^{\prime }\right)}^{T}+{\left(\bar{X},\bar{Y}\right)}^{T},$$

where **B**(⋅) is defined as

$$B\left(x\right)=\left(\begin{array}{cc} 1 & 0\\ x & \sqrt{1-x^{2}} \end{array}\right).$$

The matrix $B\left({\hat{\rho }}_{0}\right)A\left(\hat{\rho }\right)$ transforms $\left(U_{i}^{\prime },V_{i}^{\prime }\right)$ into a pair of standardized variables with zero means and unit standard deviations. Importantly, $U_{i}^{\prime }$ and $V_{i}^{\prime }$ have a correlation of ${\hat{\rho }}_{0}$. Through the following rescale and shift operations, the final variable pairs $\left(X_{i}^{\prime },Y_{i}^{\prime }\right)$ will have the same means and variances as (*X*_*i*_, *Y*_*i*_), but a correlation of ${\hat{\rho }}_{0}$ instead of $\hat{\rho }$. This ensures the sample *ρ*_*c*_ of $\left(X_{i}^{\prime },Y_{i}^{\prime }\right)$ is *ρ*_*c*(0)_. Therefore, we will use (*X* ^′^, *Y* ^′^) for the jackknife procedure for estimating $\nu_{n}^{2}$.

Occasionally, the value of ${\hat{\rho }}_{0}=\rho_{c\left(0\right)}/\hat{C}$ could be larger than 1. This will happen when $\hat{C}$ is too small but *ρ*_*c*(0)_ too large. In our implementation, we will set ${\hat{\rho }}_{0}=0.99$ in this scenario, which results in a large *p*‐value. Note that a small *C* implies large difference between *X* and *Y*, which already suggests a poor agreement. Therefore, in such cases it is unreasonable to have large *ρ*_*c*(0)_.

## 3 SIMULATIONS

### 3.1 Test for *H*_0_ : *ρ*_*c*_ = 0 versus *H*_1_ : *ρ*_*c*_ > 0

We examined the Type I error control using distributions commonly found in the literature for examining these types of test statistics across a wide range of settings.^21^, ^24^ For our simulation, we focused on testing *H*_0_ : *ρ*_*c*_ = 0 versus *H*_1_ : *ρ*_*c*_ > 0, with sample sizes *n* = 10, 25, 50, 100, 200. Each simulation utilized 10,000 Monte Carlo replications and the number of permutations used was 1000. We compared the straight large sample approximation (Asymptotic), Fisher's *Z*‐transformation (Fisher's *Z*), naive permutation test (Perm), and studentized permutation test (Stu Perm). The Type I error control for *α* = 0.05 was examined. The simulation scenarios from DiCiccio and Romano were utilized in our study:

1. Multivariate normal (MVN) with mean zero and identity covariance.

2. Exponential given as (*X*, *Y*) = *rS* ^*T*^ *u* where $S=\mathrm{diag}\left(\sqrt{2},1\right)$, *u* is uniformly distributed on the two dimensional unit circle and *r* ∼ exp(1).

3. Circular given as the uniform distribution on a two dimensional unit circle.

4. *t*_4.1_ where *X* = *W* + *Z* and *Y* = *W* − *Z*, where *W* and *Z* are i.i.d. random variables following *t*‐distributions with 4.1 degrees of freedom.

5. Multivariate *t*‐distribution (MVT) with location parameters (0, 0)^*T*^, identity covariance and 5 degrees of freedom.

The results show that for all distributions, both the untransformed and *Z*‐transformed asymptotic tests have inflated Type I error rates when *n* is small (Table 1). Although the error rates converge towards the nominal level of 0.05 as *n* increases, they only approach 0.05 when *n* ≥ 100. For MVN, the error rates are well controlled by naive permutation test, even when *n* is small. However, with other distributions, the test is either too conservative (circular) or too liberal (exponential, *t*_4.1_ and MVT), and the error rates do not converge to 0.05 as *n* increases. For example, under the *t*_4.1_ distribution, the type I error rate of naive permutation test inflates dramatically to 0.21 when *n* = 200. Meanwhile, for the circular distribution, this test becomes over conservative with a type I error rate of 0.01 when *n* = 200. On the other hand, the studentized test controls type I error rate robustly at 0.05 under all settings and even when the sample size is as small as 10. The above results demonstrated the proposed studentized permutation test is a robust method for testing *H*_0_ : *ρ*_*c*_ = 0. Although *C* = 1 in this setting, the test is not equivalent to *H*_0_ : *ρ* = 0, because the test on *ρ*_*c*_ needs to take into account the variability of $\hat{C}$.

**TABLE 1:** Type I errors for tests of *H*_0_ : *ρ*_*c*_ = 0 versus *H*_1_ : *ρ*_*c*_ > 0, when *ρ*_*c*_ = 0

Distribution	*N*	Asymptotic	Fisher's *Z*	Perm	Stu Perm
MVN	10	0.1227	0.1230	0.0489	0.0457
	25	0.0848	0.0827	0.0516	0.0518
	50	0.0677	0.0660	0.0519	0.0504
	100	0.0599	0.0587	0.0514	0.0516
	200	0.0525	0.0521	0.0479	0.0494
Exponential	10	0.1995	0.1956	0.1157	0.0608
	25	0.1410	0.1317	0.1501	0.0554
	50	0.1107	0.1037	0.1617	0.0544
	100	0.0779	0.0736	0.1619	0.0480
	200	0.0702	0.0673	0.1658	0.0517
*t*_4.1_	10	0.1665	0.1581	0.0902	0.0444
	25	0.1237	0.1123	0.1313	0.0435
	50	0.0999	0.0911	0.1552	0.0434
	100	0.0904	0.0822	0.1851	0.0487
	200	0.0788	0.0728	0.2051	0.0484
Circular	10	0.0853	0.0907	0.0184	0.0560
	25	0.0589	0.0596	0.0114	0.0473
	50	0.0534	0.0541	0.0101	0.0482
	100	0.0510	0.0512	0.0119	0.0480
	200	0.0503	0.0503	0.0117	0.0478
MVT	10	0.1579	0.1569	0.0721	0.0486
	25	0.1169	0.1114	0.0987	0.0466
	50	0.0878	0.0822	0.1076	0.0436
	100	0.0740	0.0692	0.1175	0.0435
	200	0.0703	0.0679	0.1290	0.0500

Abbreviations: MVN, multivariate normal; MVT, multivariate *t*‐distribution.

In addition to the original settings, we further examined the tests' type I error control when *C* ≠ 1. Let $\mu_{2}^{0}$ and $\sigma_{2}^{0}$ be the mean and standard deviation of *Y* in the original settings. This is achieved by either introducing a shift in *Y*, that is $\mu_{2}=\mu_{2}^{0}+2$, or having *Y* rescaled by a factor of 2, $\sigma_{2}=2\sigma_{2}^{0}$. The results are shown in supporting information, which suggest that the studentized permutation test is robust to shifts and different scales in underlying distributions.

### 3.2 Test for *H*_0_ : *ρ*_*c*_ = *ρ*_*c*(0)_ versus *H*_1_ : *ρ*_*c*_ > *ρ*_*c*(0)_

Next, we evaluated the proposed test's performance on non‐zero null hypotheses. The data was generated under the settings of *ρ*_*c*_ = 0.3 or 0.7, *μ*_2_ − *μ*_1_ = 0.5, *σ*_2_/*σ*_1_ = 1.5. The data was first generated in a similar procedure as the previous section, but was then standardized by the population standard deviations and correlated using **B**(*ρ*_*c*_/*C*) = **B**(*ρ*). The correlated data was then scaled and shifted to have desired scale and location parameters.

**FIGURE 1:**
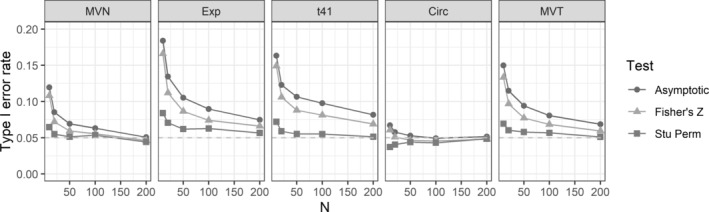
Rejection probabilities (type I error) for tests of *H*_0_ : *ρ*_*c*_ = 0.3 versus *H*_1_ : *ρ*_*c*_ > 0.3, when *ρ*_*c*_ = 0.3

**FIGURE 2:**
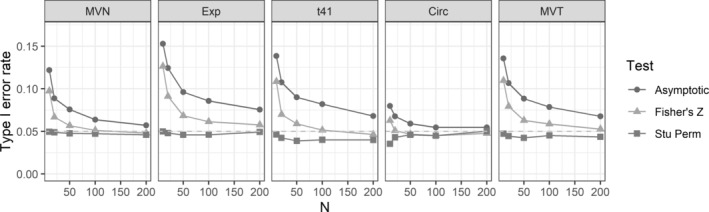
Rejection probabilities (type I error) for tests of *H*_0_ : *ρ*_*c*_ = 0.7 versus *H*_1_ : *ρ*_*c*_ > 0.7, when *ρ*_*c*_ = 0.7

The naive permutation test cannot handle a non‐zero point null, thus was not examined. Table 2 shows the type I errors for testing *H*_0_ : *ρ*_*c*_ = 0.3 and *H*_0_ : *ρ*_*c*_ = 0.7. The results are also shown graphically in Figures 1 and 2. The asymptotic test and the Fisher's *Z* test generally have inflated type I errors, where the Fisher's *Z* test shows a faster convergence to 0.05. In fact, the asymptotic test fails to achieve satisfactory type I error control even when *n* is as large as 200 except for the circular scenario. The test based on Fisher's *Z* statistic also needs *n* > 50 to achieve good type I error control in most cases. For testing *H*_0_ : *ρ*_*c*_ = 0.3 under *t*_4.1_, the Fisher's *Z* test fails to control type I error under 0.069 even when *n* = 200. On the other hand, the studentized permutation test robustly controls the type I error at desired level only with a few cases of slight inflation for testing *H*_0_ : *ρ*_*c*_ = 0.3 when *n* = 10 (Figure 1). This can be observed for exponential, *t*_4.1_ and MVT distributions. However, even in these cases, its type I error control is still superior to the competitors. For testing *H*_0_ : *ρ*_*c*_ = 0.7, the performance was robust for almost all cases (Figure 2).

**TABLE 2:** Type I errors for tests of *H*_0_ : *ρ*_*c*_ = *ρ*_*c*(0)_ versus *H*_1_ : *ρ*_*c*_ > *ρ*_*c*(0)_, where *ρ*_*c*(0)_ = 0.3 or 0.7

		*ρ*_*c*(0)_ = 0.3			*ρ*_*c*(0)_ = 0.7		
Distribution	*N*	Asymptotic	Fisher's *Z*	Stu Perm	Asymptotic	Fisher's *Z*	Stu Perm
MVN	10	0.1195	0.1082	0.0647	0.1219	0.0974	0.0495
	25	0.0853	0.0721	0.0550	0.0888	0.0668	0.0489
	50	0.0692	0.0594	0.0514	0.0757	0.0567	0.0476
	100	0.0632	0.0557	0.0535	0.0638	0.0513	0.0472
	200	0.0508	0.0461	0.0443	0.0572	0.0480	0.0460
Exponential	10	0.1839	0.1660	0.0840	0.1528	0.1264	0.0499
	25	0.1345	0.1120	0.0707	0.1243	0.0912	0.0480
	50	0.1053	0.0865	0.0620	0.0961	0.0684	0.0459
	100	0.0897	0.0741	0.0628	0.0858	0.0614	0.0461
	200	0.0747	0.0662	0.0567	0.0755	0.0577	0.0493
*t*_4.1_	10	0.1634	0.1491	0.0720	0.1385	0.1085	0.0464
	25	0.1230	0.1061	0.0590	0.1075	0.0699	0.0424
	50	0.1065	0.0880	0.0552	0.0901	0.0589	0.0389
	100	0.0977	0.0813	0.0551	0.0819	0.0515	0.0401
	200	0.0818	0.0690	0.0514	0.0680	0.0464	0.0399
Circular	10	0.0672	0.0606	0.0372	0.0798	0.0628	0.0354
	25	0.0577	0.0511	0.0406	0.0676	0.0508	0.0430
	50	0.0529	0.0464	0.0441	0.0590	0.0467	0.0460
	100	0.0493	0.0455	0.0431	0.0547	0.0452	0.0449
	200	0.0516	0.0480	0.0486	0.0547	0.0477	0.0503
MVT	10	0.1498	0.1334	0.0694	0.1357	0.1097	0.0473
	25	0.1149	0.0968	0.0604	0.1067	0.0794	0.0445
	50	0.0941	0.0773	0.0579	0.0884	0.0629	0.0424
	100	0.0806	0.0684	0.0568	0.0786	0.0587	0.0452
	200	0.0686	0.0595	0.0512	0.0676	0.0527	0.0436

*Note*: The true *ρ*_*c*_ values are 0.3 and 0.7, respectively.

Abbreviations: MVN, multivariate normal; MVT, multivariate *t*‐distribution.

**FIGURE 3:**
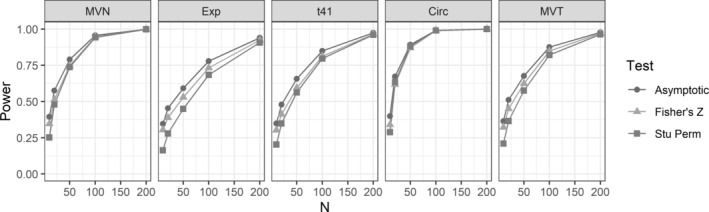
Rejection probabilities (power) for tests of *H*_0_ : *ρ*_*c*_ = 0.7 versus *H*_1_ : *ρ*_*c*_ > 0.7, when *ρ*_*c*_ = 0.8

We also investigated the power for testing *H*_0_ : *ρ*_*c*_ = 0.7 and *H*_1_ : *ρ*_*c*_ > 0.7 when the true *ρ*_*c*_ is 0.8 (Figure 3). The exact rejection probabilities are provided in Table S3. It should be noted that in most of the cases, especially when *n* ≤ 50, the powers were not comparable because Fisher's *Z* and asymptotic tests tend to have inflated type I errors. Especially, the asymptotic tests have highest power in all cases, which is due to its inflated type I errors in general. To make legitimate comparisons, we focus on the settings where the type I error of Fisher's *Z* test is <0.06. In these cases, the difference between the power of Fisher's *Z* and studentized permutation tests is generally less than 3%. For example, under the circular scenarios with *n* ≥ 25, the two tests only show negligible difference in power. Therefore, we can conclude that the proposed test robustly controls the type I error while maintains comparable power with Fisher's *Z* tests.

## 4 EXAMPLE

### 4.1 Cardiac output data

**FIGURE 4:**
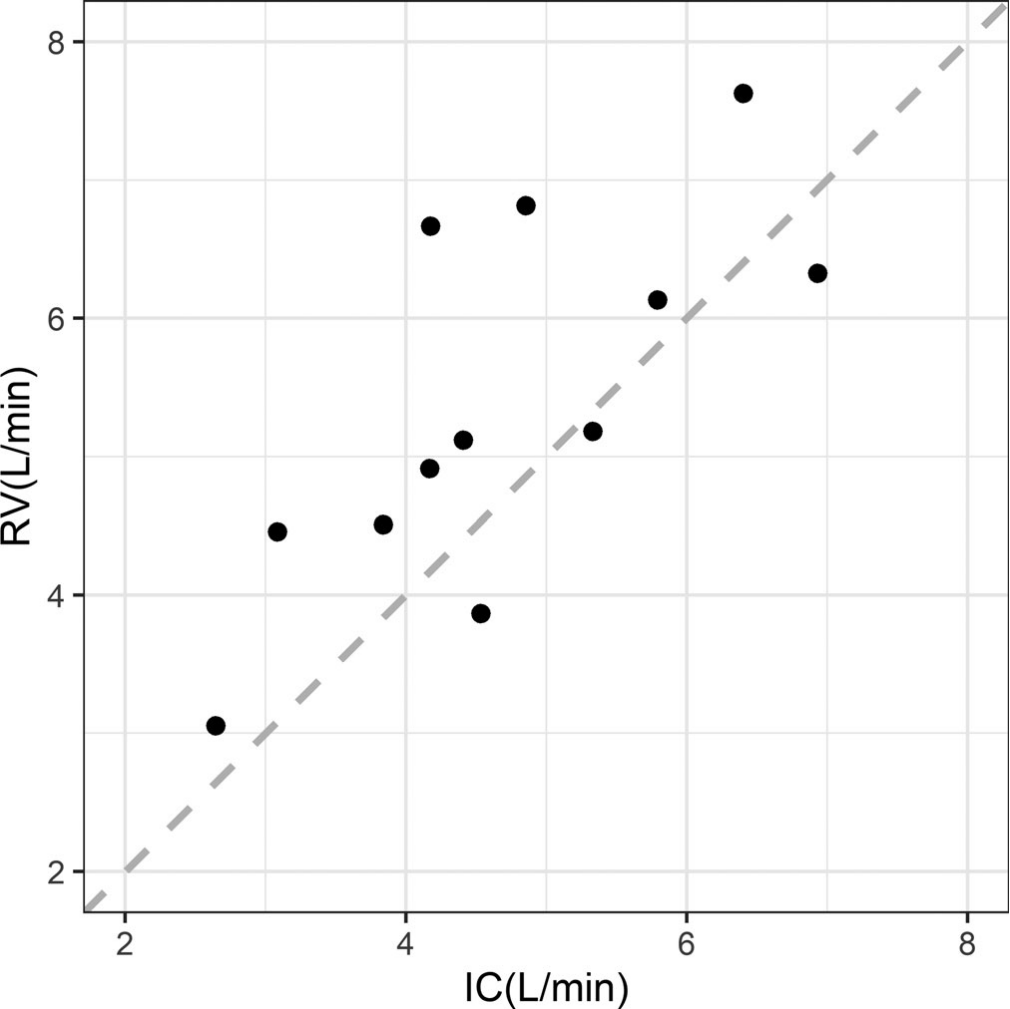
Scatter plot of cardiac output data with concordance line

As an illustration of our approach, we tested *H*_0_ : *ρ*_*c*_ = *ρ*_*c*(0)_ versus *H*_1_ : *ρ*_*c*_ > *ρ*_*c*(0)_ using cardiac output estimated from systolic time intervals based on impedance cardiography (IC) and those estimated by radionuclide ventriculography (RV) in 12 patients. The data was originally reported by Bowling et al.^25^ and is obtained from the publication by Bland and Altman.^26^ In the reported data, there are multiple pairs of observations per patient. Since modeling replicates within individuals is not within the scope of our study, we took average of the replicates for each individual, such that only one pair of averaged measurements for each individual was used for analysis. The scatter plot of the paired data is given in Figure 4. Marginal normality of data was examined by Shapiro–Wilk test, while the bivariate normality was examined by Henze–Zikler test. The *p* values of Shapiro–Wilk tests for IC and RV are 0.96 and 0.97, respectively. The *p* value of Henze–Zikler test is 0.99. Therefore, there is no evidence that the data distribution is non‐normal.

The estimated ${\hat{\rho }}_{c}$ between IC and RV is 0.64. The paired‐sample *t*‐test shows cardiac outputs estimated by RV are significantly higher than the estimates by IC (*p* = 0.026), suggesting there is a systematic difference between two techniques. The *p* values for *ρ*_*c*(0)_ = 0 and *ρ*_*c*(0)_ = 0.3 are shown in Table 3. Both permutation tests used 5000 resamples. If we are testing at level *α* = 0.05, then we will reject *H*_0_ : *ρ*_*c*_ = 0 for all tests, and conclude there is a non‐zero agreement between IC and RV in estimating cardiac outputs (Table 3). Meanwhile, we are also interested in whether there is a moderate agreement. Therefore, we tested *H*_0_ : *ρ*_*c*_ = 0.3 versus *H*_1_ : *ρ*_*c*_ > 0.3. In this case, the naive permutation test cannot be applied. Both asymptotic and Fisher's *Z* tests rejected the *H*_0_. This is a different conclusion from the studentized permutation test, which failed to reject *H*_0_. Based on the simulation results, the studentized permutation test is more reliable when the sample size is small.

**TABLE 3:** The *p*‐values of testing *H*_0_ : *ρ*_*c*_ = *ρ*_*c*(0)_ versus *H*_1_ : *ρ*_*c*_ > *ρ*_*c*(0)_ for cardiac output data

Tests	*ρ*_*c*(0)_ = 0	*ρ*_*c*(0)_ = 0.3
Asymptotic	<0.0001	0.0141
Fisher's *Z*	0.0009	0.0320
Perm	0.0038	–
Stu Perm	0.0146	0.1178

### 4.2 Echocardiographic imaging

The second example is taken from an echocardiographic imaging (EI) study.^27^ The study developed an autonomous boundary detection (ABD) algorithm to detect the limiting boundaries of the left ventricular myocardium, which requires no observer input. The study aimed to increase reliability, objectivity, and reproducibility in order to enhance the quantitative accuracy of echocardiography.

**FIGURE 5:**
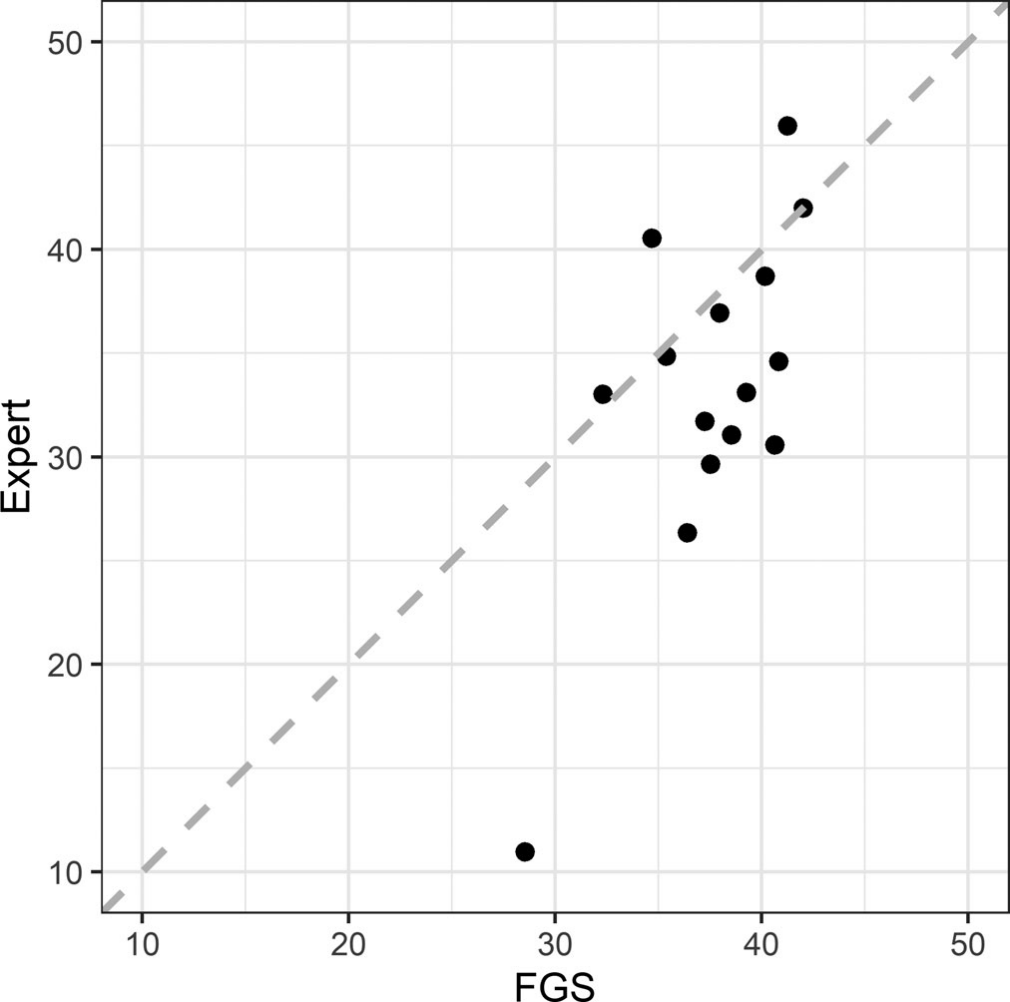
Scatter plot of echocardiographic imaging (EI) data with concordance line

Following the approach by Hutson,^13^ we have selected a subset of *n* = 15 subjects from the EI study, and use the fractional area change (FAC) as the quantity of interest. The FAC is computed as

$$\mathrm{FAC}=\frac{A_{\mathrm{ED}}-A_{\mathrm{ES}}}{A_{\mathrm{ED}}}\times 100\%,$$

where *A*_ED_ and *A*_ES_ are the endocardial areas at end diastole and end systole respectively. In this example we focus on the comparison between the FAC's as measured by the fuzzy gold standard (FGS) derived from a consensus of experts and one of the echocardiographers (Expert 2), so as to examine the tests when the agreement is poor. The agreement between FGS and the expert is visualized by a scatter plot with concordance line (Figure 5). Similarly, marginal normality of data was examined by Shapiro–Wilk test, and the bivariate normality was examined by Henze–Zikler test. The *p* values of Shapiro–Wilk tests for FGS and expert are 0.20 and 0.09, respectively. The *p* value of Henze–Zikler test is 0.09.

The paired‐sample *t*‐test shows cardiac outputs estimated by FGS are significantly higher than the estimates by the expert (*p* = 0.02) suggesting a systematic difference. From the scatter plot (Figure 5), a low agreement between two measurements was observed, and the CCC is estimated as 0.42. Table 4 shows the results for testing *H*_0_ : *ρ*_*c*_ = 0 versus *H*_1_ : *ρ*_*c*_ > 0. Similarly, both permutation tests used 5000 resamples. If we are testing at level *α* = 0.05, then only studentized permutation test failed to reject H_0_ (*p* = 0.11). All the other three tests rejected H_0_ and conclude there is a non‐zero agreement between FGS and the expert in estimating FAGS. Based on the simulation results in Section 3, the result from studentized test is more reliable and we should conclude that there is no significant agreement between FGS and the expert.

**TABLE 4:** The *p*‐values of testing *H*_0_ : *ρ*_*c*_ = 0 versus *H*_1_ : *ρ*_*c*_ > 0 for echocardiographic imaging (EI) data

Tests	*p* value
Asymptotic	0.0001
Fisher's *Z*	0.0003
Perm	0.0074
Stu Perm	0.1112

## 5 DISCUSSION

In this work, we present a robust concordance correlation permutation test for testing *H*_0_ : *ρ*_*c*_ = *ρ*_*c*(0)_. Conventional testing of the CCC relies on large sample approximations, which tends to have inflated Type I error rates when the sample size is small. This was illustrated in our simulations studies (Section 3). An alternative approach to hypothesis testing based on asymptotic approximations is to consider the corresponding permutation test. However, DiCiccio and Romano^21^ have shown that the naive permutation test of Pearson's correlation coefficient does not control type I error under non‐normality settings where two variables can be dependent but uncorrelated. Here we demonstrated that a naive permutation test for the CCC suffers a similar issue both theoretically and empirically. To solve this issue, we proposed a permutation test for the CCC based on appropriately studentized statistic following DiCiccio and Romano's approach,^21^ which controls type I error even when sample size is as small as 10 and normality assumption is violated. Importantly, while the original studentized permutation test can only handle tests of zero correlation coefficients,^24^ we further extended the studentized permutation test for more general hypotheses. Similarly, the generalized testing procedure exhibited a robust type I error control under different scenarios. The proposed method may also enable the construction of confidence sets with better coverage probability by inverting the acceptance region, which still requires future investigation. Implementation of the method is available through the R package perk (https://github.com/hyu-ub/perk).

## CONFLICT OF INTEREST

The authors declare no potential conflict of interests.

## Supporting information

**Table S1** Type I errors for tests on *H*_0_ : *ρ*_*c*_ = 0 versus *H*_1_ : *ρ*_*c*_ > 0, when $\mu_{2}=\mu_{2}^{0}+2$
**Table S2** Type I errors for tests on *H*_0_ : *ρ*_*c*_ = 0 versus *H*_1_ : *ρ*_*c*_ > 0, when $\sigma_{2}=2\sigma_{2}^{0}$
**Table S3** Power for tests on *H*_0_ : *ρ*_*c*_ = 0.7 versus *H*_1_ : *ρ*_*c*_ > 0.7, where *ρ*_*c*_ = 0.8

## Data Availability

The data that support the findings of this study are openly available.
