# Supplementary material for: A robust permutation test for the concordance correlation coefficient
Source: Pharm Stat. 2021 Feb 17;20(4):696–709. doi: 10.1002/pst.2101 (PMC8359348; doi:10.1002/pst.2101)
Supplement: Supplementary file 1 — Table S1 Type I errors for tests on H 0 : ρ c = 0 versus H 1 : ρ c > 0, when μ2=μ20+2 Table S2 Type I errors for tests on H 0 : ρ c = 0 versus H 1 : ρ c > 0, when σ2=2σ20 Table S3 Power for tests on H 0 : ρ c = 0.7 versus H 1 : ρ c > 0.7, where ρ c = 0.8 [file PST-20-696-s001.pdf]

Supplementary Materials: A Robust Permutation Test for the Concordance Correlation Coefficient

Alan D. Hutson, Han Yu

Table 1: Type I errors for tests on $H_0 : \rho_c = 0$ versus $H_1 : \rho_c > 0$, when $\mu_2 = \mu_2^0 + 2$.

Distribution	N	Asymptotic	Fisher' Z	Perm	Stu Perm
MVN	10	0.0966	0.1002	0.0487	0.0482
	25	0.0660	0.0672	0.0494	0.0477
	50	0.0601	0.0603	0.0468	0.0499
	100	0.0562	0.0567	0.0512	0.0515
	200	0.0527	0.0527	0.0508	0.0495
Exponential	10	0.1463	0.1509	0.1196	0.0602
	25	0.0961	0.0951	0.1429	0.0552
	50	0.0822	0.0815	0.1584	0.0553
	100	0.0618	0.0615	0.1636	0.0493
	200	0.0601	0.0597	0.1695	0.0512
$t_{4.1}$	10	0.1571	0.1561	0.1019	0.0493
	25	0.1067	0.1002	0.1414	0.0419
	50	0.0811	0.0750	0.1570	0.0399
	100	0.0741	0.0691	0.1823	0.0432
	200	0.0653	0.0623	0.1990	0.0445
Circular	10	0.0848	0.0860	0.0176	0.0525
	25	0.0596	0.0602	0.0126	0.0481
	50	0.0529	0.0537	0.0111	0.0486
	100	0.0499	0.0504	0.0112	0.0469
	200	0.0514	0.0515	0.0107	0.0484
MVT	10	0.1247	0.1280	0.0762	0.0502
	25	0.0894	0.0898	0.1008	0.0492
	50	0.0695	0.0684	0.1136	0.0446
	100	0.0649	0.0639	0.1205	0.0498
	200	0.0550	0.0547	0.1265	0.0445

Table 2: Type I errors for tests on $H_0 : \rho_c = 0$ versus $H_1 : \rho_c > 0$, when $\sigma_2 = 2\sigma_2^0$.

Distribution	N	Asymptotic	Fisher' Z	Perm	Stu Perm
MVN	10	0.1210	0.1273	0.0498	0.0514
	25	0.0780	0.0790	0.0507	0.0463
	50	0.0645	0.0647	0.0484	0.0507
	100	0.0598	0.0597	0.0504	0.0499
	200	0.0555	0.0552	0.0497	0.0498
Exponential	10	0.2042	0.1996	0.1200	0.0629
	25	0.1402	0.1313	0.1492	0.0566
	50	0.1039	0.0977	0.1523	0.0505
	100	0.0844	0.0793	0.1704	0.0521
	200	0.0685	0.0654	0.1641	0.0504
$t_{4.1}$	10	0.1718	0.1655	0.0989	0.0506
	25	0.1295	0.1161	0.1456	0.0463
	50	0.1053	0.0945	0.1673	0.0448
	100	0.0896	0.0807	0.1814	0.0464
	200	0.0782	0.0728	0.1967	0.0486
Circular	10	0.0815	0.0855	0.0149	0.0516
	25	0.0609	0.0622	0.0128	0.0490
	50	0.0557	0.0561	0.0115	0.0491
	100	0.0524	0.0524	0.0099	0.0489
	200	0.0500	0.0501	0.0128	0.0496
MVT	10	0.1566	0.1614	0.0754	0.0490
	25	0.1113	0.1099	0.0979	0.0487
	50	0.0954	0.0923	0.1150	0.0475
	100	0.0736	0.0714	0.1133	0.0472
	200	0.0671	0.0662	0.1237	0.0467

Table 3: Power for tests on $H_0 : \rho_c = 0.7$ versus $H_1 : \rho_c > 0.7$, when $\rho_c = 0.8$.

Distribution	N	Asymptotic	Fisher's Z	Stu Perm
MVN	10	0.3950	0.3461	0.2518
	25	0.5753	0.5140	0.4796
	50	0.7899	0.7471	0.7374
	100	0.9564	0.9459	0.9431
	200	0.9991	0.9988	0.9987
Exponential	10	0.3466	0.3048	0.1633
	25	0.4534	0.3885	0.2793
	50	0.5914	0.5300	0.4498
	100	0.7786	0.7325	0.6843
	200	0.9401	0.9240	0.9073
$t_{4.1}$	10	0.3484	0.3027	0.2032
	25	0.4786	0.4114	0.3488
	50	0.6569	0.5940	0.5627
	100	0.8497	0.8128	0.7964
	200	0.9745	0.9654	0.9603
Circular	10	0.3993	0.3402	0.2884
	25	0.6721	0.6183	0.6346
	50	0.8925	0.8701	0.8819
	100	0.9918	0.9898	0.9906
	200	> 0.9999	> 0.9999	> 0.9999
MVT	10	0.3644	0.3227	0.2095
	25	0.5112	0.4508	0.3656
	50	0.6768	0.6242	0.5756
	100	0.8753	0.8485	0.8207
	200	0.9775	0.9713	0.9640
